# Host Dependent-Transposon for a Plasmid Found in *Aeromonas salmonicida* subsp. *salmonicida* That Bears a *catB3* Gene for Chloramphenicol Resistance

**DOI:** 10.3390/antibiotics12020257

**Published:** 2023-01-27

**Authors:** Pierre-Étienne Marcoux, Sabrina A. Attéré, Valérie E. Paquet, Maude F. Paquet, Sarah B. Girard, Judith Farley, Michel Frenette, Antony T. Vincent, Steve J. Charette

**Affiliations:** 1 Institut de Biologie Intégrative et des Systèmes (IBIS), Université Laval, Quebec City, QC G1V 0A6, Canada; 2 Département de Biochimie, de Microbiologie et de Bio-Informatique, Faculté des Sciences et de Génie, Université Laval, Quebec City, QC G1V 0A6, Canada; 3 Centre de Recherche de l’Institut Universitaire de Cardiologie et de Pneumologie de Québec (IUCPQ), Quebec City, QC G1V 4G5, Canada; 4 Aquarium du Québec, Quebec City, QC G1W 4S3, Canada; 5 Groupe de Recherche en Écologie Buccale (GREB), Faculté de Médecine Dentaire, Université Laval, Quebec City, QC G1V 0A6, Canada; 6 Département des Sciences Animales, Faculté des Sciences de l’Agriculture et de l’Alimentation, Université Laval, 2425 rue de l’Agriculture, Quebec City, QC G1V 0A6, Canada

**Keywords:** pAsa-2939, antibiotic resistance gene, *Aeromonas salmonicida* subsp. *salmonicida*, plasmid, chloramphenicol, transposon

## Abstract

Plasmids that carry antibiotic resistance genes occur frequently in *Aeromonas salmonicida* subsp. *salmonicida*, an aquatic pathogen with severe consequences in salmonid farming. Here, we describe a 67 kb plasmid found in the *A. salmonicida* subsp. *salmonicida* Strain SHY15-2939 from Quebec, Canada. This new plasmid, named pAsa-2939 and identified by high throughput sequencing, displays features never found before in this bacterial species. It contains a transposon related to the Tn*21* family, but with an unusual organization. This transposon bears a *catB3* gene (chloramphenicol resistance) that has not been detected yet in *A. salmonicida* subsp. *salmonicida*. The plasmid is transferable by conjugation into *Aeromonas hydrophila*, but not into *Escherichia coli*. Based on PCR analysis and genomic sequencing (Illumina and PacBio), we determined that the transposon is unstable in *A. salmonicida* subsp. *salmonicida* Strain SHY15-2939, but it is stable in *A. hydrophila* trans-conjugants, which explains the chloramphenicol resistance variability observed in SHY15-2939. These results suggest that this bacterium is likely not the most appropriate host for this plasmid. The presence of pAsa-2939 in *A. salmonicida* subsp. *salmonicida* also strengthens the reservoir role of this bacterium for antibiotic resistance genes, even those that resist antibiotics not used in aquaculture in Québec, such as chloramphenicol.

## 1. Introduction

The Gram-negative bacterium *Aeromonas salmonicida* subsp. *salmonicida* is a major psychrophilic fish pathogen that is responsible for significant economic losses in the aquaculture industry worldwide [1]. It is the causative agent of furunculosis, a disease characterized by a high mortality and morbidity in salmonids. This disease mainly occurs in fish farming due to more stressful conditions than in the wild, which increase the risk of infection [2]. For *A. salmonicida* subsp. *salmonicida*, the type three secretion system is an essential virulence factor involved in the development of the disease and most of the genes for this system are found on a plasmid [3,4].

Antibiotics are frequently used to treat various bacterial infections in fish farms. Due to these treatments, the effectiveness of antibiotics is becoming more limited, and the emergence of multidrug-resistant pathogens is now a global concern in the aquaculture sector [5,6]. In Canada, antibiotic treatments to fight against *A. salmonicida* subsp. *salmonicida* infections are widely used in fish farms. This bacterium contains a very diverse plasmidome [7], including a large variety of plasmids that carry antibiotic resistance genes (ARGs), especially in Canadian isolates [8,9,10,11,12,13,14]. Some strains even bear plasmids that make them resistant to all antibiotics authorized by Health Canada for aquaculture, leaving the fish farmers without curative resources to fight furunculosis [10].

The rise of bacterial strains resistant to antibiotics is mainly due to the horizontal gene transfer of plasmids that carry ARGs [15]. Thus far, many plasmids found in various *A. salmonicida* subspecies that bear ARGs have been shown to be transferrable by conjugation to other bacteria from the same genus and to other genera [8,9,11,15,16]. Moreover, variants of plasmids found in other bacterial species have been observed in *A. salmonicida* subsp. *salmonicida*. Examples include pSN254b, which is an IncA/C plasmid, a variant of a plasmid found in *Salmonella enterica* [7,11].

Transposons and integrons were listed in plasmids found in *A. salmonicida* subsp. *salmonicida*. Typically, these genetic elements bear ARGs and other genes that provide diverse advantages to the bacteria [15]. The pAsa4 plasmid variants contain transposon Tn*21* with an In*2* integron [9]. A similar Tn*21* is also found in pSN254b. In this case, the transposon also includes mercury resistance genes [11]. Another frequent transposon in *A. salmonicida* subsp. *salmonicida* is Tn*1721*. Different complete or truncated forms of this transposon have been observed in plasmids like pRAS1, pAsa8 or pAsa10 [7]. This suggests that this transposon is malleable and can interact with other mobile genetic elements [17].

In this paper, using high-throughput sequencing technologies, conjugation assay, PCR genotyping and antibiotic susceptibility tests, we investigated a new plasmid named pAsa-2939, which has ARGs and is found in an *A. salmonicida* subsp. *salmonicida* strain isolated in 2015 in Québec (Canada). The analysis of this new plasmid revealed the presence for the first time in this bacterial subspecies of the *catB3* gene, which is responsible for chloramphenicol resistance. Our results demonstrate that this plasmid contains a transposon with a host-dependent instability; in *A. salmonicida* subsp. *salmonicida*, the plasmid can lose its transposon, though it has not been observed to lose its transposon in *Aeromonas hydrophila*.

## 2. Results and Discussion

The veterinary diagnostic service of the University of Montreal isolated a bacterial strain (SHY15-2939) from the kidney of a dead furunculosis-infected juvenile brook char (*Salvelinus fontinalis*) in the province of Quebec that presented an atypical antibiotic resistance profile for a strain isolated in this province. Of the six antibiotics tested by the diagnostic service, this strain exhibited resistance only to sulfadiazine/trimethoprim. Typically, in Quebec, when resistant to antibiotics, the strains that are isolated exhibit tetracycline monoresistance or multidrug resistance to two or three of the following antibiotics: Florfenicol, tetracycline and sulfadiazine/trimethoprim [8,10,11]. The other antibiotics tested by the diagnostic service are erythromycin, enrofloxacin and nalidixic acid.

To explore the ARGs further in SHY15-2939, we first used a PCR genotyping approach [10]. The *sul1* gene was the only one detected in this strain among the following list of tested genes: *cat* (found in pAsa4), *floR*, *sul1*, *sul2*, *tetA*, *tetA*(C)*, tetA*(E)*, tetH* and *tetG* [10]. This result agreed with the diagnostic service antibiogram results and suggested that there might be a new plasmid in this strain due to the atypical presence of the *sul1* gene without the other antibiotic resistance genes tested [8,9,10,11].

**Figure 1:**
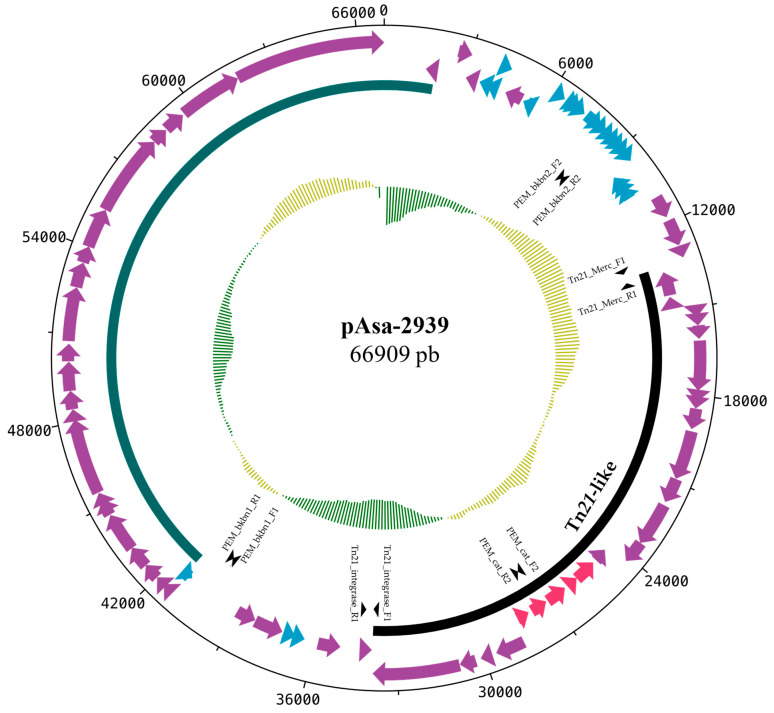
Gene map of the pAsa-2939 plasmid. This map was produced with the DNA plotter software. The red genes code for antibiotic resistance proteins, the blue genes for hypothetical proteins, and the purple genes for proteins with other known functions. The black arc indicates the transposon (Tn*21*-like). The green arc corresponds to conjugation genes and the inner circle corresponds to the GC skew. The primers used for PCR genotyping are also shown on the map with arrowheads.

To identify and characterize this potential plasmid, the genomic DNA of the SHY15-2939 strain was sequenced with Illumina technology and the reads were de novo assembled. The *sul1* gene was found on a contig with identical ends (i.e., circular). This contig was identified as a new plasmid with a complete sequence of 66.9 kb. The annotated map of this new plasmid is shown in Figure 1. Since the backbone of this plasmid does not show a strong identity with any other plasmids found in *A. salmonicida*, it was named pAsa-2939.

**Figure 2:**
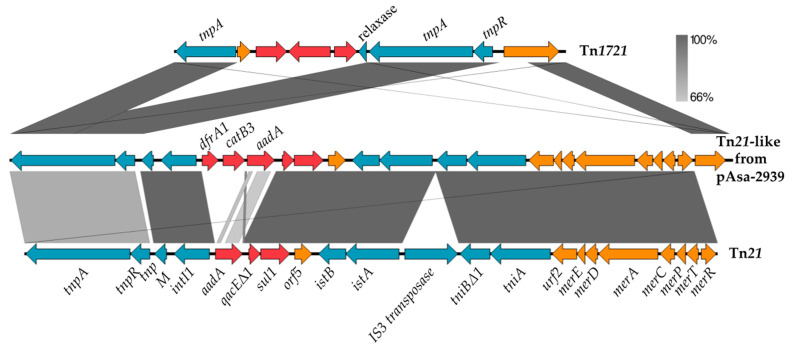
Nucleotide alignment of pAsa-2939 transposon with Tn*21* and Tn*1721*. Comparison of Tn*1721* (accession X61367) and the transposon from pAsa-2939 (Tn*21*-like) and Tn*21* (accession AF071413). The gray areas represent similar or identical segments in the sequences of the plasmids compared. The ARGs are shown in red, the mobile elements are in blue and the orange arrows represent other genes. The figure was made using Easyfig software.

A BLAST analysis revealed that a section of about 19.4 kb was very similar to the Tn*21* transposon sequences. A typical transposon from the Tn*21* family includes a gene for its own transposition, an integron, and a mercury resistance (*mer*) operon. The Tn*21* family is involved in dissemination of ARGs due to the presence of an integron [18]. This mobile element is responsible for the acquisition of various resistance genes. Figure 2 presents a comparison of pAsa-2939 transposon with Tn*21* from *E. coli* (accession AF071413). First, for the homologous regions, the sequences are highly similar with 99.97% nucleotide identity. The *tnpA* gene, which encodes a transposase, and the aminoglycoside antibiotic resistance gene *aadA* are less similar with a 78% and 57% nucleotide identity, respectively. The TnpA protein from pAsa-2939 seems to originate from the Tn*1721* with 99.19% nucleotide identity (accession X61367). Furthermore, there is a new region in the pAsa-2939 transposon which includes a chloramphenicol resistance gene and a dihydrofolate reductase. However, this transposon lacks the IS*3* family transposase in comparison with the reference sequence as shown in the Figure 2. The nearest transposon sequence to the one found in pAsa-2939 is in the pCFS3273-1 plasmid (268,665 bp, accession CP026933) from *E. coli* Strain CFS3273 (cover 89%, identity 99.87%).

Our analysis revealed the presence of a chloramphenicol resistance gene in the pAsa-2939 transposon: *catB3*. This gene is located immediately downstream from the *aatI* site of In*2*. According to the Comprehensive Antibiotic Resistance Database (CARD), this gene is widely found in many bacterial species [19]. Based on an analysis on 26 November 2022 on the NCBI Nucleotide collection (nr/nt) database, the *catB3* gene was found with 100% identity, 100% coverage in 57 strains of *Aeromonas* sp. and in another 10 strains of *Aeromonas* sp. with a coverage and identity between 98.42% and 99.84%. These strains are mainly mesophile *Aeromonas* sp. (*hydrophila*, *caviae*, *veronii*, etc.). In the case of *A. salmonicida* sp., the *catB3* gene is also found in plasmids from *A. salmonicida* sp. (pS44-1, pS121-1a, pS121-1b) and *A. salmonicida* subsp. *masoucida* (pAsmA and pA). Interestingly, for these five plasmids, the *catB3* gene is part of a transposon that is quite similar to the one found in pAsa-2939. This gene has never been identified in the past in strains of *A. salmonicida* subsp. *salmonicida,* according to sequence databases.

A PCR genotyping screen was performed on 225 *A. salmonicida* subsp. *salmonicida* strains isolated in Quebec (Table S1). The tested strains were isolated from fish sick with furunculosis in the years from 2001 to 2020. The primer pairs used in this screen targeted the *catB3* gene and two regions of the pAsa-2939 backbone (Table S2 and Figure 1). Strain SHY15-2939 was used as a positive control in these analyzes while Strain 01-B526, whose genome is fully known and does not have this resistance gene, was the negative control [20]. None of the 225 strains tested gave a positive result for the plasmid itself or the *catB3* gene. This shows that, of the strains tested so far in Quebec, Strain SHY15-2939 is the only known example of an *A. salmonicida* subsp. *salmonicida* strain that bears this plasmid and the *catB3* gene.

Until now, genes that confer resistance to chloramphenicol in *A. salmonicida* subsp. *salmonicida* have only been found in the pAsa4, pAsa4c and pAsa7 plasmids. All these plasmids were found in strains from Europe. pAsa4 and pAsa4c bear the same *cat* gene while pAsa7 has a different version of the *cat* gene [4,9,21]. The *catB3* gene found in the SHY15-2939 strain would therefore be the first example of a chloramphenicol resistance gene found in Quebec in *A. salmonicida* subsp. *salmonicida*. This in itself is surprising considering that chloramphenicol (and its salts and derivatives) is on the list of banned drugs for use on any food-producing animal since 1997, thus not used in aquaculture in Canada [22]. The antibiotic most used in aquaculture in Quebec is florfenicol, an antibiotic of the same class as chloramphenicol. However, the *cat* genes (found in pAsa4 and pAsa7 [9,21]) provide specific resistance to chloramphenicol compared to the *floR* gene, which is very often found on plasmids such as pSN254b in strains from Quebec and which confers resistance to both florfenicol and chloramphenicol [23,24].

*A. salmonicida* subsp. *salmonicida* has often been proposed to be an ARG reservoir, even for ARGs of little use to the bacterium itself. We recently described this situation for the pRAS3 plasmids increasingly found in strains of *A. salmonicida* subsp. *salmonicida* in Quebec and which confer resistance to tetracycline, an antibiotic with limited use in aquaculture, but widely used in the pork industry in the same province [14].

Chloramphenicol is a naturally occurring, broad-spectrum antibiotic that is artificially manufactured. This antibiotic causes various negative side effects for human health [25]. Consequently, the use of this antibiotic is limited to the treatment of a variety of infections in companion animals and for life-threatening infections in humans [25,26]. Despite limited use, a study on organic antibiotics detected chloramphenicol in surface waters and sewage plant effluent [27]. Thus, it is possible that fish are exposed to chloramphenicol even if its use is banned in food-producing animals [25]. The presence of pAsa-2939 in *A. salmonicida* subsp. *salmonicida* in waterways is an example of the One Health concept where human and animal health and the environment are intertwined.

Based on a BLAST analysis, the conjugative genes predicted in pAsa-2939 (Figure 1) do not seem to derive from any other *A. salmonicida* plasmids. The closest result was the pWP8-S18-ESBL-04 plasmid (accession AP022255.1) found in *Aeromonas caviae* with an identity percentage of 92.36% with a BLASTN analysis. Interestingly, this plasmid also shares the second-highest identity with the pAsa-2939 backbone (84.55%). The plasmid with the highest identity with the pAsa-2939 backbone is the *A. caviae* pWP2-W18-ESBL-01_1 plasmid (accession AP021928.1) with an identity value of 89.05%. However, the identity between pWP8-S18-ESBL-04 and pAsa-2939 has more coverage (63%) than pWP2-W18-ESBL-01_1 (56%).

We verified the functionality of the pAsa-2939 conjugative genes. A strain of *A. hydrophila* (HER1210) and a strain from *E. coli* (DH5α) were used as recipient bacteria for these conjugation assays (Table 1). Two attempts were made with each of the bacteria. While the conjugation in *A. hydrophila* worked very well on both occasions, giving dozens of conjugants, the transfer did not work at all in the case of *E. coli* DH5α. This strain of *E. coli* was found to be a good recipient bacterium for the pRAS1b plasmid from the donor strain 2004-072 [13], which was used as a control to show that the method used was adequate. The presence of the plasmid and of the *catB3* gene was confirmed by PCR in the trans-conjugants of *A. hydrophila* using the primers described above (Table S2). This result is likely not surprising since, when performing a BLAST analysis on the nr/nt database, that the top 50 plasmids sharing the highest identity with pAsa-2939 backbone were found only in *Aeromonas* sp. In the case of pRAS1b, plasmids from many different genera are found in the top 50 sequences that share identity with this plasmid, suggesting a greater host range of the backbone of pRAS1b than the backbone of pAsa2939. Thus, we can consider that pAsa-2939 is a plasmid with a narrower host range. Since the replicon type and some specific genes predominately determine the host range of a plasmid [28], we can conclude that pAsa-2939 does not contain genetic features compatible with many bacterial hosts and seems to be specific to the *Aeromonas* genus.

We then evaluated the chloramphenicol minimal inhibitory concentration (MIC) for both SHY15-2939 and an *A. hydrophila* trans-conjugant with appropriate controls. While it was possible to repeatedly obtain an MIC of 256 µg/mL in *A. hydrophila* possessing pAsa-2939 (<16 µg/mL for the same strain without the plasmid, *n* = 3), it was not possible to determine the MIC accurately for Strain SHY15-2939. Even after six attempts, this gave variable results ranging from <16 to 256 µg/mL with a median at 96 µg/mL (Table 2). Considering that we could not accurately determine the MIC for chloramphenicol for SHY15-2939, this suggests that the plasmid or a part of the plasmid is probably unstable in this strain, though it is not unstable in *A. hydrophila* HER1210 used as a conjugation recipient strain.

To address this point, we explored two different approaches: (1) See if the plasmid becomes permanently lost in SHY15-2939 with a long period of cultivation and (2) analyze the read frequency in the raw Illumina sequencing data of the plasmid compared to the chromosome reads as a reference.

**Figure 3:**
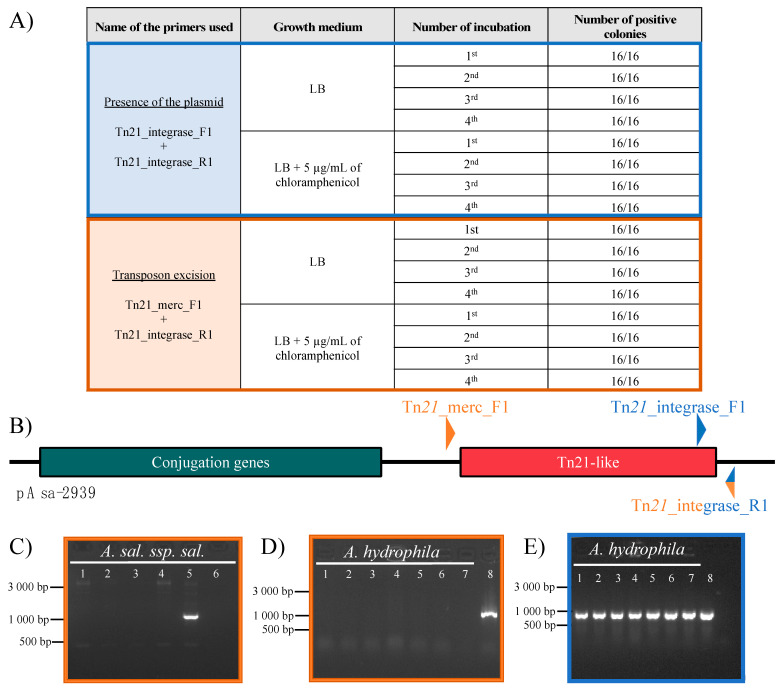
Transposon instability in SHY15-2939 confirmed by PCR analysis. (**A**) Analysis of different lysates from *A. salmonicida* subsp. *salmonicida* SHY15-2939 grown on different medium using different primers. This confirms the excision of the transposon in a part of the bacterial population (in orange) while the majority of the bacteria keep an intact plasmid (in blue). (**B**) Schematic representation of pAsa-2939 found in *A. salmonicida* subsp. *salmonicida* SHY15-2939. The region in green corresponds to the gene involve in the conjugation of the plasmid and the *Tn21*-like is represented by the region in red. The primers are shown in blue or orange depending on the combination used for the PCR. The Tn21_integrase_R1 primer was used in both PCR reaction. (**C**) Confirmation of the specificity of the Tn21_Merc_F1 and Tn21_integrase R1 primers (orange combination) for transposon excision. Strains tested are in this order from lane 1 to 6: 01-B526, 01-B516, A449, SHY16-3432, SHY15-2939 and water as a negative control. (**D**) The PCR result from 7 trans-conjugants of *A. hydrophila* with the pAsa-2939 plasmid (line 1 to 7) using the primers Tn21_Merc_F1 and Tn21_integrase R1 (orange combination). The eighth well corresponds to the positive control which is *A. salmonicida* ssp. *salmonicida* SHY15-2939. (**E**) A PCR amplification of the primers Tn21_integrase_F1 and Tn21_integrase_R1 (blue combination) for *A. hydrophila* with pAsa-2939 on the same samples than in D.

To evaluate the plasmid stability in SHY15-2939, we first incubated the bacteria at 18 °C on two LB media, one without antibiotic and the other containing 5 µg/mL of chloramphenicol. Then we produced PCR lysates for 16 colonies from each medium and streaked those colonies on new LB agar without antibiotics. We streaked the colonies three times (for a total cultivation period of 288 h) and produced a total of 64 lysates. As shown in the Figure 3A,B, the PCR primers targeting junction between the Tn*21*-like transposon and the plasmid show a signal for every colony. Thus, the variability of the MIC is not due to the loss of the plasmid. Since the plasmid seems to be stable, the variability of the MIC might be due to the instability of the transposon. To verify this, we used a pair of primers that are located outside the sequence of the transposon. When the strain carries pAsa-2939 without the transposon, an amplification is produced by the excision of the transposon and the junction of the two plasmid ends together. We confirmed this result by testing this primer pair on other *A. salmonicida* subsp. *salmonicida* strains not carrying pAsa-2939 (Figure 3C). None of them gave an amplicon confirming the excision of the transposon of pAsa-2939 in SHY15-2939. It appeared that an amplicon can be produced with this pair of primers for Strain SHY15-2939 regardless the growth conditions or the time of cultivation (Figure 3A,B). On the other hand, no amplicon where detected with the same primers with the *A. hydrophila* trans-conjugants that bears pAsa-2939 suggesting no transposon excision in this case (Figure 3D,E). The amplicon obtained with SHY15-2939 was sequenced, which revealed that it corresponds to the expected sequence for the junction of the plasmid after excision of the transposon. These results confirm the capacity of the transposon to be excised from the plasmid in Strain SHY15-2939 and the stability of the transposon in *A. hydrophila* trans-conjugant. These results validate the difference in chloramphenicol sensitivity observed for SHY15-2939 and the *A. hydrophila* trans-conjugant.

**Figure 4:**
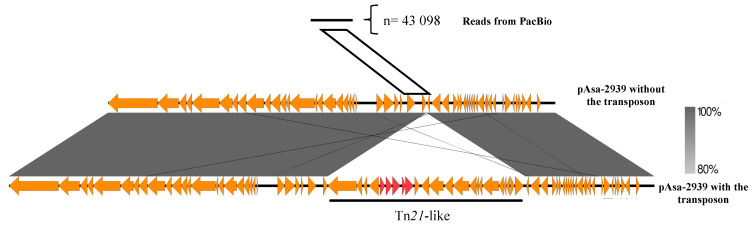
Transposon instability in SHY15-2939 confirmed by bioinformatics analysis. Comparison between a read from PacBio and the sequence of pAsa-2939 without the transposon (theoretical) and the pAsa-2939 plasmid including the transposon. The gray area represents similarities between the two plasmid versions. The red arrows represent the ARGs and the orange arrows represent other genes. The white parallelogram represents the region used to detect PacBio reads corresponding to the junction consequent to transposon excision.

To confirm the presence of the plasmid without the transposon, the genomic DNA of Strain SHY15-2939 was sequenced with long-read sequencing (PacBio SMRT technology). After performing the assembly, it was possible to again obtain the complete assembled sequence of the pAsa-2939 plasmid. To confirm the potential absence of the transposon in a proportion of cells, the reads from the PacBio sequencing were mapped on a theoretical version of pAsa-2939 without the transposon. In total, 43 098 reads were aligned to the junction using minimap2 [31] (Figure 4), which again confirms the excision of the transposon. It was not possible to find a secondary shorter assembly of the plasmid that would be the truncated version of the plasmid without the transposon. This is not surprising considering how the assembly algorithm works and that there were a low number of reads that were mapped on the junction of pAsa-2939 without the transposon.

Using the Illumina reads, it appeared that the coverage was lower for the region of the transposon than for the rest of the plasmid (Table 3). The difference between these two values is prominent, suggesting that about 50% of the plasmid molecules do not bear the transposon. When compared to the read coverage of the chromosome, pAsa-2939 seems to be only in one copy in the cells. The coverage of the Tn*21*-like transposon is less, which suggests that a sub-population of the bacteria bears pAsa-2939 with the transposon, and another sub-population bears the same plasmid without the Tn*21*-like transposon. The dataset used for this analysis was produced from bacteria grown without antibiotic.

To our knowledge, this is the first time that transposon instability has been observed in *Aeromonas* (we did not find any results through Web of Science research), though transposon instability has been described for many transposons in other bacterial species [32,33,34]. It is important to keep in mind that *catB3* is located in the In*2* portion of Tn*21*. One study showed that integrons could excise and reorganize the order of the cassettes located downstream of the *aatI* site [35]. Using this mechanism, the Tn*21* transposon could reposition cassettes that are still part of the mobile genetic element but with a decreased or even almost null level of expression. Another possibility would be the complete excision of the entire Tn*21* or of the In*2*. This type of rearrangement was already detected in other bacteria [18,36] and would explain the differences in coverage observed between the replicon backbone and the Tn*21*-corresponding sequences (Table 3).

## 3. Materials and Methods

### 3.1. Bacterial Strains

The pAsa-2939 plasmid was found in psychrophilic *A. salmonicida* subsp. *salmonicida* Strain SHY15-2939 isolated in 2015 from the kidney of a dead furunculosis-infected juvenile brook char (*Salvelinus fontinalis*) in the province of Quebec. SHY15-2939 was provided by the diagnostic service of the Faculty of Veterinary Medicine of the University of Montreal. The other strains used in this study are described in Table 1 and Table S1. They were grown from the frozen stocks on furunculosis agar (FA) for 3 days at 18 °C as previously described [30]. *E. coli* DH5α and *A hydrophila* were grown from the frozen stock on tryptic soy agar (TSA) (Wisent, St-Bruno, QC, Canada) for one day at 37 °C.

### 3.2. PCR Analyses

DNA lysates of each strain were obtained using a previously described protocol [17]. PCR analyses were first performed to detect the presence of ARGs against chloramphenicol/florfenicol (*cat* and *floR*), sulfonamide (*sul1* and *sul2*) and tetracycline (*tetA*, *tetA*(C)*, tetA*(E)*, tetH* and *tetG*) in Strain SHY15-2939 prior genomic sequencing using a previously described multiplex PCR approach [10]. A PCR screening was performed to detect the new plasmid and the new chloramphenicol resistance gene in 225 *A. salmonicida* subsp. *salmonicida* strains (Table S1). To evaluate the plasmid stability, we performed a PCR that targeted the backbone of pAsa-2939. The PCR cycle was as follow: 2 min 30 s at 95 °C, 30 cycles of 30 s at 95 °C, 30 s at 55 °C, and an elongation step 15 s at 68 °C, ending by a 5 min extension at 68 °C. We also designed a primer pair for each extremity of the transposon to verify the excision of this mobile element. The amplicons produced by these primer pairs amplify a region corresponding to the insertion site of the transposon with the flanking regions in the backbone of the plasmid and the transposon. Then, we used primers located outside the transposon to investigate the junction that resulted from the excision. All new primers used in this study are listed in Table S2. The PCR cycle for these primers was slightly different with an elongation time of 1 min 15 s at instead of 15 s.

### 3.3. DNA Extraction and Sequencing

The total genomic DNA of Strain SHY15-2939 was extracted using DNeasy Blood and Tissue kits (Qiagen, Montreal, QC, Canada) with the addition of an RNase A treatment step (20 µg/mL, Ambion, ThermoFisher, Mississauga, ON, Canada) according to the manufacturer’s protocol. Sequencing libraries were prepared from purified bacterial DNA using the Nextera XT DNA Library Preparation Kit (Illumina, San Diego, CA, USA) and the sequencing was performed using a MiSeq instrument system (Illumina, San Diego, CA, USA) at the Plateforme d’Analyse Génomique of the Institut de Biologie Intégrative et des Systèmes (Université Laval, Quebec City, QC, Canada).

### 3.4. Sequence Assembly and Analyses

The sequencing reads were de novo assembled using A5-miseq version 20,160,825 [37]. Then CONTIGuator version 2.7.5 was used for mapping the contigs on the reference genome of Strain A449 (NC_009348.1). The unmapped contigs were screened using BLAST [38] to find the *sul1* gene. It was found on a 66 909 bp contig, which was annotated using Prokka [39]. Its sequence was deposited in DDBJ/ENA/GenBank under accession number OQ067800. The plasmid sequence was then further characterized using BLAST [38], and verified by Artemis version 16.0.0 [40]. Finally, the plasmid maps were created using DNAPlotter version 18.0.0 [41] and EasyFig version 2.2.2 [42]. For the alignment, the reads were mapped on the junction of pAsa-2939 using minimap2 (v2.24). Then the mapped reads were analyzed using BLAST (v2.13.0+) to find the reads overlapping the junction. To evaluate the coverage of the Illumina reads we used the program bwa (0.7.17). The statistical analyses were done using Qualimap (v2.2.2) [43].

### 3.5. PacBio Sequencing

The total genomic DNA was also sequenced using a Pacific BioScience RS II system (PacBio, San Francisco, CA, USA) at the Génome Québec Innovation Center (McGill University, Montreal, QC, Canada). The assembly and the circularization of the contigs were done using Unicycler version 0.4.7 [44].

### 3.6. Bacterial Conjugation Assays

Bacterial conjugation assays were performed as previously described [9] to transfer pAsa-2939 from the donor strain (SHY15-2939) to *A. hydrophila* strain HER1210 (ATCC 7966) and *E. coli* Strain DH5α (ATCC 29552). A conjugation assay was also performed between a donor strain bearing pRAS1b (2004-072) and the same recipient strains as a control (Table 1) [13]. PCR analyses were performed to detect the presence of pAsa-2939 in trans-conjugants using the primers described above. The absence of *A. salmonicida* subsp. *salmonicida* among the trans-conjugants was confirmed by primers that target the *tapA* gene [45] (Table S1). Conjugation assays were performed twice with each recipient strain.

### 3.7. Assessment of the MIC

The bacteria were inoculated on LB agar (Wisent, St-Bruno, QC, Canada) directly from frozen stocks and were grown at 18 °C for 72 h for *A. salmonicida* subsp. *salmonicida* strains and at 37 °C for 24 h for *A. hydrophila* HER1209 before each experiment. Several colonies of each isolate were suspended in fresh LB broth (Wisent, St-Bruno, Qc, Ca) and the OD_600_ was adjusted to 0.1 which represents 1.3 × 10^8^ CFU/mL [46]. The chloramphenicol and florfenicol MICs of the strains were determined using a previously described protocol [11] with minor modifications of the bacterial growth conditions performed at 18 °C and 37 °C for 24 h for mesophilic strains and only at 18 °C for 72 h for psychrophilic strains. Every assay was performed at least in technical duplicate and three biological replicates for *A. hydrophila* and 6 biological replicates for *A. salmonicida* subsp. *salmonicida* strains.

### 3.8. Stability of the Plasmid

Strain SHY15-2939 was inoculated on LB agar and LB+5 µg/mL of chloramphenicol at 18 °C. After 3 days, 16 lysates from 16 distinct colonies were produced for the PCR experiment. Then 16 colonies were streaked on the new medium (LB or LB+5 µg/mL of chloramphenicol) according to media used for the initial growth from the frozen stock and incubated at 18 °C again. In total, 48 lysates for each medium were produced for the PCR analyses. We also wanted to investigate the stability of the transposon for the *A. hydrophila* trans-conjugant that bears pAsa-2939. Therefore, *A. hydrophila* strain was grown three times at 37 °C for 24 h on LB agar. Each time, a PCR lysate originating from a colony was produced. Theses lysates were tested for the presence of pAsa-2939 and transposon excision by PCR genotyping.

## 4. Conclusions

This study, which began with a simple observation of a new plasmid in a strain of *A. salmonicida* subsp. *salmonicida* isolated in Quebec (pAsa-2939), made it possible to highlight a new resistance gene (*catB3*) never before observed in this bacterial subspecies. The plasmid that carries *catB3* has an unstable transposon in its original strain, SHY15-2939, but this plasmid is stable in *A. hydrophila*. This result and the similarity of pAsa-2939 to other plasmids found in mesophilic *Aeromonas* strains suggest that the acquisition of pAsa-2939 by SHY15-2939 is potentially a fortuitous event and that the true host of the ancestor of this plasmid was an *Aeromonas* sp. that shares the same ecological niche. The mechanisms explaining the instability of the transposon are unknown for the moment and could be a good focus for future research; identification of potential variants of this plasmid would be useful to understand its instability. Regardless of how the pAsa-2939 plasmid arrived in a strain of *A. salmonicida* subsp. *salmonicida*, the results of our study once again demonstrate the great reservoir potential of this bacterial subspecies to carry ARGs, including resistance to an antibiotic not even used in aquaculture and the connection of this subspecies with its community for the exchange of mobile genetic elements.

## Figures and Tables

**Table 1:** Strains used in this study.

Strain Name	Species	Lifestyle	Commentary	Reference
SHY15-2939	*A. salmonicida* subsp. *salmonicida*	Psychrophilic	Bears pAsa-2939	This study
01-B526	*A. salmonicida* subsp. *salmonicida*	Psychrophilic	Negative control for MIC experiments	[29]
JF3791	*A. salmonicida* subsp. *salmonicida*	Psychrophilic	Bears pAsa7, positive control for MIC experiments	[21]
HER1210	*Aeromonas hydrophila*	Mesophilic	Recipient strain for conjugation experiments	ATCC 7966
DH5α	*Escherichia coli*	Mesophilic	Recipient strain for conjugation experiments	ATCC 29552
2004-072	*A. salmonicida subsp. salmonicida*	Psychrophilic	Bears pRAS1b, positive control for conjugation test	[13]
01-B516	*A. salmonicida* subsp. *salmonicida*	Psychrophilic	Negative control for the stability of pAsa-2939	[30]
A449	*A. salmonicida* subsp. *salmonicida*	Psychrophilic	Negative control for the stability of pAsa-2939	[4]
SHY16-3432	*A. salmonicida* subsp. *salmonicida*	Psychrophilic	Negative control for the stability of pAsa-2939	[8]

**Table 2:** MIC of chloramphenicol at 18 °C for *A. salmonicida* subsp. *salmonicida* and at 37 °C for *A. hydrophila*.

Strain Name	Species	MIC (µg/mL)						Concentration (µg/mL)
		n1	n2	n3	n4	n5	n6	256
SHY15-2939	*A. salmonicida* subsp. *salmonicida*	128	128	<16	32	256	64	128
01-B526	*A. salmonicida* subsp. *salmonicida*	<16	<16	<16	<16	<16	<16	64
JF3791	*A. salmonicida* subsp. *salmonicida*	256	256	256	256	256	256	32
HER1210	*A. hydrophila*	<16	<16	<16				16
HER1210 + pAsa-2939	*A. hydrophila*	256	256	256				0

**Table 3:** Read coverage in the raw sequencing of SHY15-2939.

Name	Length (pb)	Mapped Bases	Mean Coverage	Standard Deviation
Chromosome	4,614,604	457,980,600	99.25	70.0
TTSS	33,204	3,739,690	112.63	42.9
Tn*21*-like	20,530	900,231	43.85	14.4
pAsa-2939 without the transposon	46,380	3,889,492	83.86	59.0

## Data Availability

The pAsa-2939 sequence was deposited in DDBJ/ENA/GenBank under the accession number OQ067800.

## Supplementary Materials

The following supporting information can be downloaded at: https://www.mdpi.com/article/10.3390/antibiotics12020257/s1, Table S1. *A. salmonicida* subsp. *salmonicida* strains analyzed for the presence of pAsa-2939 and *catB3* gene; Table S2. Primers used in this study.
