# Supplementary material for: Host Dependent-Transposon for a Plasmid Found in Aeromonas salmonicida subsp. salmonicida That Bears a catB3 Gene for Chloramphenicol Resistance
Source: Antibiotics (Basel). 2023 Jan 27;12(2):257. doi: 10.3390/antibiotics12020257 (PMC9952659; doi:10.3390/antibiotics12020257)
Supplement: Supplementary file 1 [file antibiotics-12-00257-s001.zip › antibiotics-2161089-supplementary.pdf]

Supplementary material

Host Dependent-Transposon for a Plasmid Found in *Aeromonas salmonicida* subsp. *salmonicida* that bears a *catB3* Gene for Chloramphenicol Resistance

Pierre-Étienne Marcoux[1,2,3], Sabrina A. Attéré[1,2,3], Valérie E. Paquet[1,2,3], Maude F. Paquet[1,2], Sarah B. Girard[1,2], Judith Farley[4], Michel Frenette[2,5], Antony T. Vincent[6] and Steve J. Charette[1,2,3*]

1. Institut de Biologie Intégrative et des Systèmes (IBIS), Université Laval, Quebec City, QC, Canada, G1V 0A6

2. Département de biochimie, de microbiologie et de bio-informatique, Faculté des sciences et de génie, Université Laval, Quebec City, QC, Canada, G1V 0A6

3. Centre de recherche de l'Institut universitaire de cardiologie et de pneumologie de Québec (IUCPQ), Quebec City, QC, Canada, G1V 4G5

4. Aquarium du Québec, Quebec City, QC, Canada, G1W 4S3

5. Groupe de Recherche en Écologie Buccale (GREB), Faculté de médecine dentaire, Université Laval, Quebec City, QC, Canada, G1V 0A6

6. Département des sciences animales, Faculté des sciences de l'agriculture et de l'alimentation, Université Laval, 2425, rue de l'Agriculture, Quebec City, QC, Canada G1V 0A6

[*]To whom correspondence should be addressed: Institut de Biologie Intégrative et des Systèmes (IBIS), Pavillon Charles-Eugène-Marchand, 1030 avenue de la Médecine, Université Laval, Quebec City, QC, Canada, G1V 0A6. ORCID : 0000-0002-0199-2852.
Steve.charette@bcm.ulaval.ca; Telephone: 1-418-656-2131, ext. 406914

Table S1. *A. salmonicida* subsp. *salmonicida* strains from Quebec (Canada) and analyzed for the presence of pAsa-2939 and *catB3* gene.

Strain name	Year isolated	Origin (fish)	Strain name	Year isolated	Origin (fish)
01-B522	2001	Brook trout	SHY15-2743	2015	Rainbow trout
01-B526	2001	Brook trout	SHY15-1998	2015	Brook trout
01-B516	2001	Brook trout	SHY15-2405	2015	Brook trout
07-9324	2007	Brook trout	SHY15-2407	2015	Brook trout
07-7817	2007	N/A	SHY15-2461	2015	Atlantic salmon
07-7346	2007	Atlantic salmon	SHY15-2816	2015	Brook trout
07-5957	2007	Atlantic salmon	SHY15-2951	2015	Brook trout
08-2647	2008	Brook trout	SHY15-4166	2015	Brook trout
09-0167	2009	Atlantic salmon	SHY15-4688	2015	Brook trout
07-7287	2007	Brook trout	SHY15-5108	2015	Brook trout
08-2783	2008	Brook trout	SHY15-3738	2015	Brook trout
08-4188	2008	Brook trout	SHY15-4029	2015	Brook trout
5093-3	N/A	Brook trout	SHY15-3379	2015	Brook trout
5093-4	N/A	Brook trout	SHY15-3412	2015	Brook trout
5490-1	N/A	Brook trout	SHY15-3138	2015	Brook trout
5490-2	N/A	Brook trout	SHY15-3292	2015	Brook trout
5480-4	N/A	Brook trout	SHY15-2950	2015	Brook trout
5490-5	N/A	Brook trout	SHY16-010	2016	Brook trout
5704-1	N/A	Brook trout	SHY16-1761	2016	Rainbow trout
5704-2	N/A	Brook trout	SHY16-1976	2016	Brook trout
5704-4	N/A	Brook trout	SHY16-2128	2016	Brook trout
5704-5	N/A	Brook trout	SHY16-2419	2016	Artic char
5704-6	N/A	Brook trout	SHY16-2777	2016	Brook trout
5704-3	N/A	Brook trout	SHY16-2889	2016	Atlantic salmon
M17524-09	2009	Brook trout	SHY16-3012	2016	Brook trout
M14349-09	2009	Atlantic salmon	SHY16-3244	2016	Rainbow trout
M23281-09	2009	Brook trout	SHY16-3269	2016	Rainbow trout
M23067-09	2009	Brook trout	SHY16-3430	2016	Brook trout
M19438-09	2009	Brook trout	SHY16-3431	2016	Brook trout
M16583-09	2009	Brook trout	SHY16-3421	2016	Brook trout
M14231-09	2009	Atlantic salmon	SHY16-3432	2016	Brook trout
M11743-09	2009	Brook trout	SHY16-3636	2016	Brook trout
M11431-09	2009	Brook trout	SHY16-3705	2016	Brook trout
M10419-09	2009	Brook trout	SHY16-3707	2016	Rainbow trout
M9906-09	2009	Brook trout	SHY16-3941	2016	Brook trout
M9954-10	2010	Brook trout	SHY16-3985	2016	Salmon
M10935-11	2011	Brook trout	SHY16-4170	2016	Lake trout
M15448-11	2011	Brook trout	SHY16-4280	2016	Brown trout
M16474-11	2011	Brook trout	SHY17-1999	2017	Atlantic salmon

M19878-11	2011	Brook trout
M11500-11	2011	Brook trout
M16486-11	2011	Brook trout
M13460-11	2011	Brook trout
M13729-11	2011	Brook trout
M14481-11	2011	Brook trout
M15879-11	2011	Brook trout
M17739-11	2011	Brook trout
M13732-11	2011	Brook trout
M17053-11	2011	Brook trout
M15878-11	2011	Rainbow trout
M13182-11	2011	Atlantic salmon
M17735-11	2011	Brook trout
M15576-11	2011	Brown trout
M15469-11	2011	Brook trout
M22710-11	2011	Brook trout
M13764-11	2011	Brook trout
M18076-11	2011	Lumpfish
M23911-11	2011	Brook trout
M12357-12	2012	Brook trout
M21375-12	2012	Brook trout
M16237-12	2012	Brook trout
M12976-12	2012	Brook trout
M22895-12	2012	Brook trout
M10745-12	2012	Brown trout
M9754-12	2012	Brook trout
M17930-12	2012	Brook trout
M12418-12	2012	Brook trout
M21368-12	2012	Brook trout
M13050-12	2012	Brook trout
M24783-12	2012	Brook trout
M13566-12	2012	Brook trout
M14404-12	2012	Atlantic salmon
M16671-12	2012	Brook trout
M16042-12	2012	Brook trout
SHY13-162	2013	Brook trout
SHY13-574	2013	Brook trout
SHY13-1470	2013	Brook trout
SHY13-2188	2013	Brook trout
SHY13-2222	2013	Brook trout
SHY13-2257	2013	Brook trout
SHY13-2263	2013	Brook trout
SHY13-2317	2013	Brook trout

SHY17-2000	2017	Lake trout
SHY17-2815	2017	Brook trout
SHY17-3069	2017	Rainbow trout
SHY17-3278	2017	N/A
SHY17-3309	2017	N/A
SHY17-3542	2017	Brown trout
SHY17-4280	2017	Brown trout
SHY17-4800	2017	Brown trout
SHY17-5108	2017	N/A
SHY18-2489	2018	Brook trout
SHY18-2492	2018	Brown trout
SHY18-2645	2018	Atlantic salmon
SHY18-2984	2018	Atlantic salmon
SHY18-3199	2018	Atlantic salmon
SHY18-3218	2018	Brown trout
SHY18-3221	2018	Brown trout
SHY18-3337	2018	Brown trout
SHY18-3388	2018	Atlantic salmon
SHY18-3658	2018	Brown trout
SHY18-3759	2018	Brown trout
SHY18-3777	2018	Arctic char
SHY18-4069	2018	Brown trout
SHY18-4395	2018	Brown trout
SHY18-5532	2018	Brown trout
SHY19-1070	2019	Brown trout
SHY19-3453	2019	Brown trout
SHY19-3598	2019	Brown trout
SHY19-3661	2019	Arctic char
SHY19-3662	2019	Brown trout
SHY19-3931	2019	Brown trout
SHY19-3931	2019	Brown trout
SHY19-3932	2019	Brown trout
SHY19-4656	2019	Brown trout
SHY19-4655	2019	Brown trout
SHY19-4654	2019	Brown trout
SHY19-4738	2019	Brown trout
SHY19-2939	2019	Brown trout
SHY16-005	2016	Brown trout
SHY16-619	2016	Brown trout
SHY16-2560	2016	Brown trout
SHY16-2951	2016	Rainbow trout
SHY16-3196	2016	Brown trout
SHY16-3325	2016	Brown trout

SHY13-2425	2013	Brook trout
SHY13-2458	2013	Brook trout
SHY13-2534	2013	Atlantic salmon
SHY13-2627	2013	Brook trout
SHY13-2630	2013	Brook trout
SHY13-2825	2013	Brook trout
SHY13-2873	2013	Brook trout
SHY13-2909	2013	Brook trout
SHY13-3101	2013	Brook trout
SHY13-3127	2013	Brook trout
SHY13-3795	2013	Brook trout
SHY13-3798	2013	Brook trout
SHY13-3799	2013	Brook trout
SHY14-2246	2014	Brook trout
SHY14-4161	2014	Brook trout
SHY14-3290	2014	Brook trout
SHY14-3402	2014	Brook trout
SHY14-2420	2014	Brook trout
SHY14-1503	2014	Brook trout
SHY14-710	2014	Brook trout
SHY14-2996	2014	Arctic char
SHY14-2939	2014	Brook trout
SHY14-3674	2014	Brook trout
SHY14-3502	2014	Brook trout
SHY14-2928	2014	Brook trout
SHY14-2485	2014	Brook trout
SHY15-1459	2015	Rainbow trout
SHY15-1543	2015	Brook trout
SHY15-1846	2015	Brook trout
SHY15-1847	2015	Arctic char
SHY15-2589	2015	Arctic char

SHY16-3359	2016	Brown trout
SHY16-3524	2016	Brown trout
SHY16-3841	2016	Brown trout
SHY16-4939	2016	Brown trout
SHY17-2625	2017	Brown trout
SHY17-3034	2017	Brown trout
SHY17-3487	2017	Brown trout
SHY17-3572	2017	Brown trout
SHY17-4092	2017	Brown trout
SHY17-4105	2017	Brown trout
SHY19-3597	2019	Brown trout
SHY19-3863	2019	Brown trout
SHY19-4071	2019	Brown trout
SHY17-3070-2	2017	Brown trout
SHY20-1481	2020	Brown trout
SHY20-1590	2020	Brown trout
SHY20-2575	2020	Brown trout
SHY20-2715	2020	Brown trout
SHY20-2897	2020	Brown trout
SHY20-3058	2020	Rainbow trout
SHY20-3059	2020	Brown trout
SHY20-3116	2020	Brown trout
SHY20-3274	2020	Brown trout
SHY20-3312	2020	Brown trout
SHY20-3406	2020	Artic char
SHY20-3522	2020	Brown trout
SHY20-3753	2020	Brown trout
SHY20-3808	2020	Brown trout
SHY20-4100	2020	Brown trout
SHY20-5455	2020	Brown trout

N/A : information not available.

Table S2. Primer used in this study for genotyping

Primer	Sequence 5′ – 3′	Amplicon size (bp)	Reference
catB3 gene			
PEM_cat_F2	CCAATTCCACCACTCCATCTC	306	This study
PEM_cat_R2	GGACCGTGATGACGTTGATAAG		
pAsa2939 backbone			
PEM_bkbn1_F1	TGACCAACGCTAAAGGGATAAA	294	This study
PEM_bkbn1_R1	ACCAGGCAAAGGCTCAATAA		
PEM_bkbn2_F2	CCGACCTGATTTGTGACCTATT	239	This study
PEM_bkbn2_R2	ACATCGGCCCGTTCTATTTC		
Verification of the transposon			
Tn21_intégrase_F1	TGGGAGCACATCAACCTAAC	657	This study
Tn21_intégrase_R1	GCCTCACAGAAGCGCTAT TA		
Tn21_Merc_F1	GCTGGCATACTGTCTCTATCAC	775	This study
Tn21_Merc_R1	CGACATGAACGCGCAAATAG		
